# High Silent Prevalence of Zinc Deficiency and Impaired Immunity in Children Under Five Years of Age Admitted to a Regional Referral Hospital in Uganda

**DOI:** 10.7759/cureus.74816

**Published:** 2024-11-30

**Authors:** Keneth Junior Male, Barnabas Atwiine, Gertrude N Kiwanuka

**Affiliations:** 1 Department of Biochemistry, Mbarara University of Science and Technology, Mbarara, UGA; 2 Department of Pediatrics and Hematology and Oncology, Mbarara University of Science and Technology, Mbarara, UGA

**Keywords:** immunity, interleukins, lymphocytes, under 5 children, white blood cell, zinc deficiency, zinc status

## Abstract

Introduction

Zinc deficiency (ZnD) impairs the development of acquired immunity and contributes to growth failure in children under five years of age. However, the prevalence of ZnD and its association with immunity in this age group in Uganda have not been well explored. This study aimed to determine the prevalence of ZnD and explore the associations between low serum zinc levels and total white blood cell count, differential cell counts, and levels of IL-1 and IL-2 in children aged 12 to 59 months.

Methods

In this cross-sectional study, we enrolled children aged 12 to 59 months upon admission to the pediatrics ward of Masaka Regional Referral Hospital (MRRH), located in Masaka City, Southern Uganda. Anthropometric measurements were taken and interpreted using the WHO growth standards charts for age and sex. Whole blood cell counts, serum zinc levels, CRP, and IL-2 and IL-4 were measured. Student’s t-test, Mann-Whitney test, and correlation coefficients were used to assess relationships between variables.

Results

A total of 40 children (mean age 27.8 (SD 10.6) months; 50% boys) were enrolled. Nearly a third (13/40) of the children were malnourished (22.5% stunted and 12.5% wasted), and 82.5% had anemia (Hb <11.0 g/dL). The prevalence of ZnD was 40.6%. Serum zinc levels showed a positive correlation with total white blood cell count (r_s_ = 0.41, p = 0.02) and lymphocyte count (r_s_ = 0.43, p = 0.01). However, no association was found between ZnD and levels of IL-2 or IL-4.

Conclusions

The study revealed a high prevalence of ZnD, with serum zinc levels correlating with both total white blood cell and T cell counts, but not with IL-2 levels, in children under five years of age at the time of admission. We recommend the routine inclusion of ZnD assessment and treatment in the care of sick children in the region. Additionally, a larger multicenter longitudinal study is needed to further evaluate the association between malnutrition and health outcomes in this age group.

## Introduction

Zinc is an essential element involved in numerous cellular processes, particularly in the normal growth, development, and function of both innate and adaptive immune cells [1]. Since the body does not store zinc, it must be consumed daily through the diet to meet the needs of all individuals [2]. Without sufficient intake, zinc deficiency (ZnD) occurs. In developing countries, where diets are primarily plant-based, zinc intake is often insufficient, leading to a high prevalence of ZnD [3]. In Uganda, ZnD is reported to affect 20-70% of the population [4].

In children under five years of age, ZnD is linked to repeated infections and weakened immunity. Malaria, pneumonia, and diarrhea, all of which negatively impact growth, have been associated with ZnD [5]. The WHO recommends supplementing all children under five years with zinc during episodes of diarrhea [6]. This recommendation is supported by clinical evidence demonstrating that zinc supplementation reduces the frequency of infections, such as pneumonia and diarrhea, and promotes better growth [7-9]. However, zinc supplementation has not been widely adopted, even in regions with a high prevalence of deficiency. In Uganda, routine standard care for healthy children does not include zinc supplementation to prevent morbidity [10], and ZnD assessments are not routinely conducted.

Given the high estimated prevalence of ZnD in certain regions, assessing and appropriately managing ZnD in a timely manner is crucial. Therefore, this study aimed to investigate the prevalence of ZnD (as measured by serum zinc levels) and its association with immune function in children under five years at a regional referral hospital.

## Materials and methods

Study design and setting

We conducted a hospital-based cross-sectional study at the admission unit of the pediatric ward of Masaka Regional Referral Hospital (MRRH), located in Masaka City, Southern Uganda, between December 2021 and January 2022. MRRH is situated 132 km southwest of the capital city, Kampala, and serves eight districts, with a catchment area of over two million people and an annual admission of up to 24,000 patients. 

Sample size estimation

We estimated the sample size of 40 participants using the Krejcie and Morgan table [11] for a finite population, based on an average of 45 children aged 12 to 59 months admitted to the Pediatrics Ward at MRRH each month. The calculation assumed a 95% CI and a population proportion of 0.5.

Study population and sampling method

We consecutively enrolled a sample of 40 children aged 12 to 59 months, of both sexes, whose guardians provided formal consent for participation in the study. Children who were very ill or had received zinc as a treatment for diarrhea or therapeutic feeds (including F-75, F-100, diluted therapeutic milk, and ready-to-use therapeutic food) in the past 28 days were excluded from the study.

To avoid disrupting the admission process or the routine care of the children on the ward, we implemented two key measures: (1) an additional 2 ml of blood was collected by the clinical team during the routine blood draw, so that phlebotomy was performed only once, and (2) written consent was obtained after the child had received initial treatment at the admission unit, when required.

Data collection procedures

The study adhered to the principles outlined in the Declaration of Helsinki and received ethics review and approval from the Mbarara University of Science and Technology Research Ethics Committee (MUST-REC; reference number 21/01-19). It also obtained administrative clearance from MRRH. Guardians of all study participants provided written consent prior to enrolling their children in the study.

Data on the children’s sex, age, and anthropometric measurements (weight and mid-upper arm circumference (MUAC)) were collected. To assess the participants’ nutritional status, we measured height, weight, and MUAC in accordance with the Uganda Ministry of Health’s Integrated Management of Acute Malnutrition guidelines [12]. Weight was measured to the nearest 0.1 kg using a calibrated weighing scale (model Seca 874, Germany), and length/height was measured to the nearest 0.1 cm using an infantometer for children under 24 months and a stadiometer for those above 24 months. Z-scores based on WHO child growth standards [13] were recorded to assess nutritional status. A non-stretching pediatric MUAC tape was used for MUAC measurement. The clinical diagnoses at admission were also recorded.

Blood samples were collected from the forearm or dorsum of the hand vein of each child. A total of 5 mL was drawn, with 2 mL transferred to an EDTA anticoagulant-coated tube and 3 mL into a free-anticoagulant tube. Whole blood samples were analyzed for full blood count within 30 minutes of collection using an automated hematology analyzer (Sysmex XN-350, Sysmex, Asia Green, Singapore) to measure the number of innate immune cells. Inflammation was assessed by measuring serum CRP levels using an automated biochemical analyzer (Architect c4000, Abbott, Canon Medical Systems Corporation, Tochigi, Japan).

Serum zinc levels were analyzed using flame atomic absorption spectrophotometry (200 Series AAS, Agilent Technologies, California, USA). To study the adaptive immune response, T-cell cytokines (IL-2 for T helper 1 cells and IL-4 for T helper 2 cells) were analyzed using enzyme-linked immunosorbent assay (ELISA) kits from MyBiosource.com (San Diego, USA), following the manufacturer’s guidelines. IL-2 and IL-4 ELISA samples were run in duplicates, and serum zinc was analyzed in triplicates.

Data analysis

Continuous variables (age, MUAC, serum zinc levels, cell counts, and IL levels) are presented as means ± SDs or medians with IQRs, as appropriate. Categorical variables, such as gender, are presented as percentages. We used the Student’s t-test and Mann-Whitney U test to assess differences between categories, depending on the data distribution. Pearson’s correlation coefficient or Spearman’s rank correlation was used, as appropriate, to assess correlations between serum zinc levels (the predictor variable) and white blood cell counts (total count and differentials), IL-2, and IL-4 levels (outcome variables). A significance level of p < 0.05 was considered. All statistical analyses were performed using IBM SPSS Statistics for Windows, Version 20.0 (Released 2011; IBM Corp., Armonk, NY, USA).

## Results

Study profile

**Figure 1:**
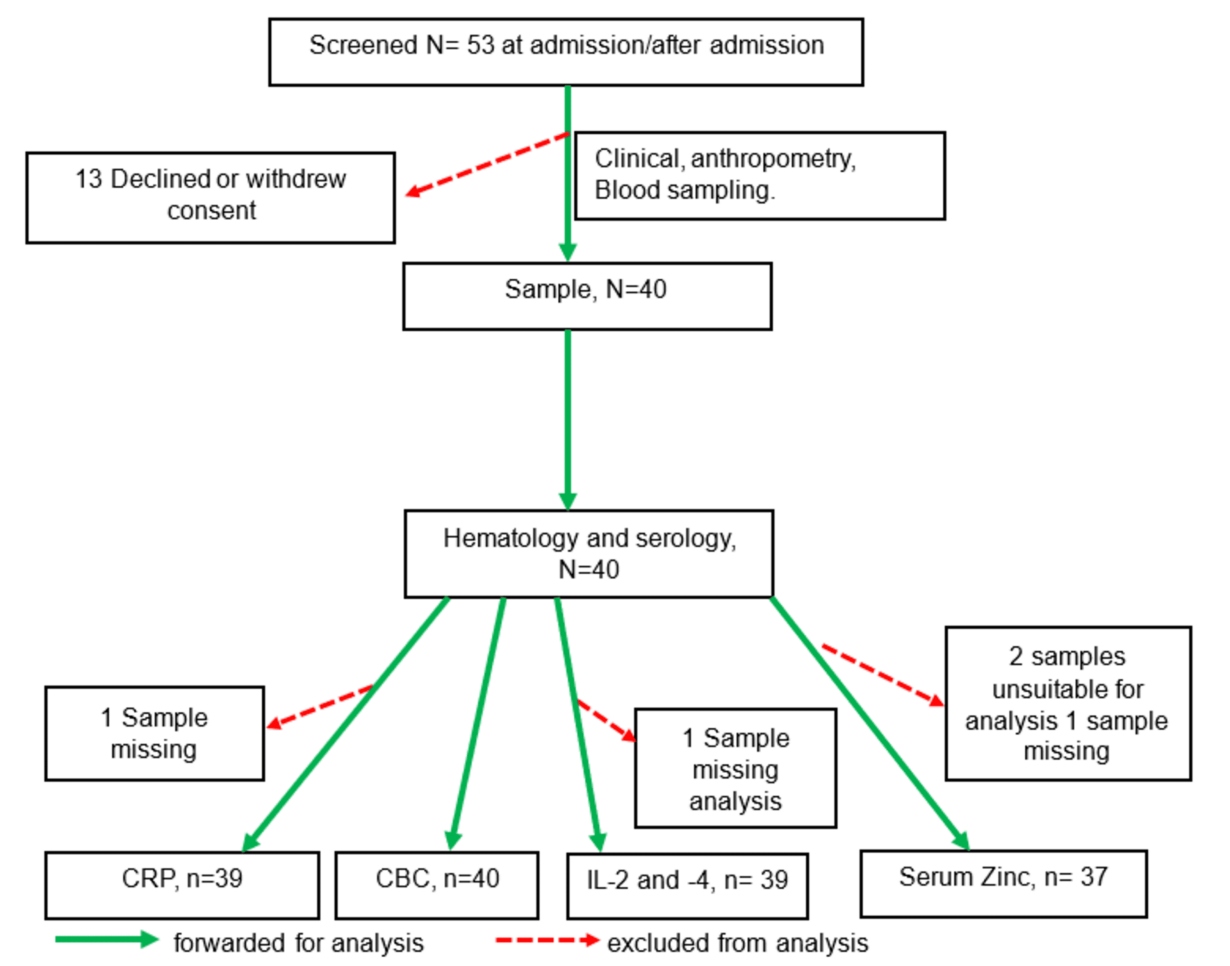
Study flowchart The illustration outlines the procedures for participant recruitment and data collection.

A total of 53 children met the study inclusion criteria during the enrollment period. However, the guardians of 13 children declined to provide consent. As a result, 40 children and their guardians were successfully recruited into the study (Figure 1).

Demographics and anthropometry of the study participants 

The study recruited an equal number of males and females. The median age was 28.9 months (IQR 17.6-35.7), with girls being older than boys (p = 0.0029, t = 3.182). The proportion of stunted children was 22.5% (nine out of 40), and 12.5% (five out of 40) were wasted. There were no significant differences in the demographic and anthropometric characteristics between the two genders of participants (Table 1).

**Table 1:** Demographic and anthropometry characteristics of study participants p-values represent tests for differences in characteristics and were calculated using ^*^Student’s t-test and ^#^Mann-Whitney U test. MUAC, mid-upper arm circumference

Characteristic	All participants (n = 40)	Male (n = 20)		Female (n = 20)	p-value	
Median age, months (IQR)	28.9 (17.6, 35.7)	19.87 (15.4, 40.0)		33.86 (24.9, 38.4)	0.0032^*^	
Median weight, kg (IQR)	10.6 (8.2, 12.0)	10.10 (8.22, 11.8)		11.0 (7.9, 12.9)	0.665^*^	
Median height, cm (IQR)	86.0 (74.5, 93.0)	84.0 (74.7, 92.9)		87.4 (73.4, 93.5)	0.794^#^	
Median MUAC, cm (IQR)	15.1 (13.7, 16.0)	15.2 (14.1, 15.9)		15.0 (13.0, 16.0)	0.6487^*^	
Number of children stunted, (%)	9 (22.5)	4 (20)		5 (25)	NA	
Number of children wasted (%)	5 (12.5)	2 (10)		3 (15)	NA	

In addition, 80% (32/40) of the study participants were diagnosed with the three most common diseases associated with ZnD: malaria, pneumonia, and diarrhea. The remaining eight children were diagnosed with septicemia (n = 3), febrile convulsions (n = 2), or severe anemia (n = 3).

White blood cell counts

Overall, participants had a low lymphocyte count, with a median of 3.70 (IQR 2.87-5.66), and a high monocyte count, with a median of 1.44 (IQR 0.68-2.61) (Table 2).

**Table 2:** White blood cell counts of the study participants ^* ^Laboratory-established reference ranges

White blood cell parameters (× 10^3^μL)	Mean (± SD)	Median (IQR)	Reference range^*^
White blood cell count	12.97 (±7.854)	10.61 (7.52, 16.14)	6.0-17.5
Neutrophils	6.17 (±4.61)	4.67 (2.7, 8.31)	1.5-8.5
Lymphocytes	4.692 (±3.209)	3.70 (2.87, 5.66)	4.0-13.5
Monocytes	1.732 (±1.327)	1.44 (0.68, 2.61)	0.2-0.9
Eosinophils	0.086 (±0.121)	0.05 (0.003, 0.13)	0.0-0.5
Basophils	0.293 (±0.797)	0.05 (0.04, 0.1)	0.0-0.3

In addition, 60% (24/40) of the participants had lymphopenia, and 67.5% (27/40) had monocytosis. Furthermore, 82.5% (33/40) of the participants had low hemoglobin concentrations (normal range: 11-14 g/dL), and 37.5% (15/40) had a low reticulocyte count (normal range: 3.7-5.3 × 10³ cells/μL).

Serum parameters

The median serum zinc level was 64.3 μg/dL (IQR 47.3, 70.1). Considering a normal range for serum zinc of 57-110 μg/dL [14], the prevalence of ZnD was 40.5% (15/37). The study participants had high levels of CRP, with a median CRP level of 43.2 g/L (IQR 22.9, 94.3), well above the normal range of <5 g/dL, as well as elevated IL levels. No gender-based differences were observed in the levels of the serum parameters studied (Table 3).

**Table 3:** Study participants’ serum parameters p-values represent tests for differences in characteristics and were calculated using ^*^Student’s t-test and ^#^Mann-Whitney U test.

Serum parameter	All participants	Male	Female	p-value
Median zinc, μg/dL (IQR)	64.3 (47.3, 70.1)	65.9 (50.3, 69.3)	60.9 (46.9, 81.3)	0.73^#^
Median CRP, g/dL (IQR)	43.2 (22.9, 94.3)	35.4 (22.9, 83.0)	56.1 (22.4, 161.2)	0.55^#^
Median IL-2, pg/mL (IQR)	129.6 (92.5, 244.4)	155.9 (101.6, 567.7)	111.4 (74.8, 166.6)	0.07^#^
Median IL-4, pg/mL (IQR)	93.8 (79.4, 115.6)	86.6 (80.6, 102)	99.1 (78.6, 120)	0.22^*^

Association of serum zinc levels with white blood cell counts and ILs

Serum zinc levels were positively and significantly correlated with both total white blood cell count (r_s_ = 0.41, p = 0.02) and lymphocyte count (r_s_ = 0.43, p = 0.01). In contrast, IL-2 levels showed a weak positive correlation with serum zinc levels, although this was not statistically significant (r_s_ = 0.30, p = 0.09) (Table 4).

**Table 4:** Correlations between serum zinc and white blood cells, CRP, and ILs among study participants p-values represent correlation tests and were calculated using Spearman’s rank correlation and ^*^Pearson’s moment correlation. The Th1/Th2 ratio was calculated by dividing IL-2 levels by IL-4 levels.

Parameter	Correlation	p-value
White blood cell	0.41	0.02
Neutrophils	0.27	0.14
Lymphocytes	0.43	0.01
Monocytes	0.13	0.5
Eosinophils	-0.001	0.96
Basophils	0.34	0.06
CRP	0.12	0.5
IL-2	0.3	0.09
IL-4	0.05	0.8^*^
Th1/Th2 ratio	0.11	0.5

Notably, there were no significant differences in white blood cell counts, serum CRP levels, or cytokine levels between children with normal zinc levels and those who were zinc deficient at the time of admission.

## Discussion

We aimed to determine the prevalence of ZnD and assess its association with immune function in children aged 12 to 59 months admitted to the pediatric ward of MRRH. Our findings reveal a high prevalence of ZnD (40.5%, 15/37) among the study population. Additionally, we identified significant correlations between serum zinc levels and both total white blood cell count and T cell counts during acute illness. This highlights a gap in micronutrient deficiency prevention, as participants were not evaluated for ZnD despite showing signs of it.

The high prevalence of ZnD in this study is consistent with previous reports, with a prevalence ranging from 20% to 70% in children under five years old [4]. For example, a study among rural preschool-aged children in South Africa reported a 42.5% prevalence of ZnD [15], and a pooled analysis in sub-Saharan Africa found prevalence rates of 35%, 40%, and 63% in Ethiopia, South Africa, and Nigeria, respectively [16]. These similarities may be attributed to shared risk factors such as poor health services, low dietary zinc intake, and frequent diarrhea.

While 40.5% of participants were zinc-deficient, only 22.5% were stunted, suggesting that the ZnD observed was likely acute. The prevalence of stunting (22.5%) in our study is lower than the national rate of 29% [4], pointing to both a potential community-level ZnD and an ongoing public health concern for a country striving to achieve Sustainable Development Goal 3 by 2030 [17]. This high prevalence underscores the need for preventive services in Uganda, where zinc supplementation is not yet integrated into routine care.

Our study also revealed low lymphocyte counts, with significant positive correlations between serum zinc and both total white blood cell count (r_s_ = 0.41, p = 0.02) and lymphocyte count (r_s_ = 0.43, p = 0.01), suggesting that serum zinc is crucial for immune cell proliferation and growth. Previous studies have shown that ZnD leads to a reduction in peripheral blood immune cells, including lymphocytes [18], which may explain the observed low lymphocyte counts. This relationship has not been extensively studied in clinical settings, particularly in children under five years of age. The high prevalence of anemia among participants, often co-occurring with ZnD, further suggests inadequate zinc intake, as reported in earlier studies from developing countries [19,20].

Interestingly, the study participants had elevated neutrophil and monocyte counts, which may reflect a maintained response of the myeloid lineage in the face of acute illness. This finding is consistent with a study by Ibrahim et al., which examined the effects of childhood malnutrition on host defenses [21]. During infection or inflammation, tissue-resident cells, such as neutrophils and monocytes, are mobilized quickly from tissues like bone marrow and the spleen to replenish circulating levels [22,23]. This mechanism may have contributed to the preservation of these cell populations in the early stages of illness in our study participants. Additionally, 80% of participants had malaria, pneumonia, and/or diarrhea, diseases commonly associated with ZnD, indicating a potentially impaired immune system due to acute ZnD.

Assessment of T cell cytokines provides insights into immune system activation. In this study, we evaluated the association between serum zinc levels and the activity of Th1 and Th2 T cell subsets by measuring their predominant cytokines. The levels of IL-2 and IL-4 cytokines were higher in our study compared to a previous study by Sciuca and Neamtu [24], who reported lower IL levels in children with community-acquired pneumonia (CAP) and wheezing. The difference could be due to the fact that the participants in the earlier study had a single diagnosis of CAP, whereas many participants in our study had multiple diagnoses, most involving infections. This may have led to a more robust or overwhelming immune response in our study population.

The elevated CRP levels observed in our study further suggest significant inflammation. Similarly, high IL-2 and IL-4 levels have been reported in children with severe CAP [25], indicating that severe infection, like that seen in our study, is associated with elevated cytokine levels. Interestingly, IL-2 levels were consistently higher than IL-4 in our study, which could reflect the pleiotropic roles of IL-2 in activating pro-inflammatory cytotoxic T cells and natural killer cells, as well as stimulating the release of interferon-gamma (IFN-γ) and anti-inflammatory T regulatory cells during immune responses [26].

ZnD is known to alter cytokine production by the Th1 and Th2 subsets, impairing Th1 cytokine synthesis (e.g., IFN-γ and IL-2), while Th2 cytokines (e.g., IL-4, IL-6, and IL-10) may remain unaffected [27]. In a study by Sciuca and Neamtu [24], the Th1/Th2 ratio in children with CAP was 0.7, suggesting a Th2 predominance. However, in our study, the Th1/Th2 ratio was greater than 1 (2.6), indicating Th1 predominance and a highly inflammatory state, further supported by neutrophilia and monocytosis. This is in contrast to previous studies that reported reduced Th1 cytokine production in ZnD [28], possibly due to a longer follow-up period in those studies [29]. The differences in findings may reflect the acute nature of ZnD observed in our study.

Lastly, our correlation analysis between serum zinc and CRP levels did not show a significant relationship, as seen in some related studies. This may be due to the stage of infection [30], before zinc redistribution takes effect, or the relatively small sample size in our study.

Limitations

Our study was limited by several factors. Firstly, the relatively small sample size compared to other studies, as data were collected during the COVID-19 pandemic, may have affected the robustness of the findings. Secondly, the cross-sectional nature of the cohort design prevented us from establishing causality. Additionally, since the study was conducted at a single center, the results may not be fully generalizable to a broader patient population.

Recommendations

Assessment and treatment of ZnD should be integrated into the comprehensive management of micronutrient deficiencies. Longitudinal studies with larger sample sizes, conducted across multiple centers, are needed to better understand the associated factors and to develop effective assessment tools for ZnD.

## Conclusions

Our cross-sectional study determined the prevalence of ZnD and its association with impaired immunity in children admitted to a regional referral hospital in Uganda. The findings revealed a high, silent prevalence of ZnD, which was linked to compromised immunity, particularly among children under five years of age at admission. We recommend incorporating the assessment and treatment of ZnD into the routine care of sick children in the region. Additionally, we suggest conducting a larger multicenter longitudinal study to evaluate the relationship between undernutrition, clinical outcomes, and the duration of hospital stay in this age group.
